# “Doctor, my back hurts and I cannot sleep.” Depression in primary care patients: Reasons for consultation and perceived depression stigma

**DOI:** 10.1371/journal.pone.0248069

**Published:** 2021-03-05

**Authors:** Ines Heinz, Sabrina Baldofski, Katja Beesdo-Baum, Susanne Knappe, Elisabeth Kohls, Christine Rummel-Kluge

**Affiliations:** 1 Department of Psychiatry and Psychotherapy, Medical Faculty, University Leipzig, Leipzig, Germany; 2 German Alliance Against Depression, Leipzig, Germany; 3 Behavioral Epidemiology, Institute of Clinical Psychology and Psychotherapy, Technische Universität Dresden, Dresden, Germany; 4 Center for Clinical Epidemiology and Longitudinal Studies, Technische Universität Dresden, Dresden, Germany; Chiba Daigaku, JAPAN

## Abstract

**Background:**

General practitioners (GPs) play a significant role in depression care. Recognition of depression is crucial for adequate treatment but is impeded by a high portion of depressed patients only reporting physical symptoms to their GP. Among the many reasons for this phenomenon is mental health stigma. We investigated how patients with depression differed from patients without depression regarding the types and number of complaints presented to their GP, as well as their depression stigma. For the subgroup of patients with depression, potential associations between perceived depression stigma and number and types of presented complaints were investigated to see if these might reflect the patient’s intention to conceal mental health symptoms due to fear of being stigmatized by others. Further, we investigated if perceived depression stigma is related to depression treatment.

**Methods:**

Data on depressive symptoms (assessed by the Depression Screening Questionnaire; DSQ), depression stigma (assessed by the Depressions Stigma Scale; DSS), type of complaints reported to the GP and treatment-related factors were collected from 3,563 unselected primary care patients of 253 GPs in a cross-sectional epidemiological study (“VERA study”) in six different German regions. Data of a total of 3,069 patients was used for analysis on complaints reported to the GP (subsample of the VERA study), and for 2,682 out of 3,069 patients data on a stigma questionnaire was available.

**Results:**

Nearly half of the primary care patients with depression (42.2%) reported only physical complaints to their GP. Compared to patients without a depression diagnosis, patients with depression reported twice as many complaints to their GP with a mean of 2.02 (1.33) vs. 1.2 (0.69), including a more frequent combination of physical and mental symptoms (28.8% vs. 3.5%). Patients with depression showed higher total stigma compared to patients without depression, *Mdn =* 48 (*IQR* 40–54) vs. *Mdn =* 46.3 (*IQR* 29–53), due to higher perceived stigma, *Mdn =* 27 (*IQR* 21–32) vs. *Mdn =* 25.9 (*IQR* 20–29). Perceived stigma was associated with male gender (beta -.14, *p* = .005) and a lack of pharmacological treatment (beta -.14, *p* = .021) in patients with a depression diagnosis.

**Conclusion:**

The number of complaints presented to the GP might function as a marker to actively explore depression in primary care patients, in particular when both physical and mental symptoms are reported. Perceived depression stigma should also be addressed especially in male patients. Further research should clarify the role of perceived stigma as a potential inhibitor of pharmacological treatment of depression in primary care.

## Introduction

Depression is among the leading causes for disability-adjusted life years [1]. Effective and evidence-based treatments are available [2], nevertheless more than 50% of patients do not receive depression-specific treatment [3, 4], or else experience delays to treatment due to a variety of reasons [5, 6]. Among them are both structural and financial barriers, as well as a low perceived need for professional help and fear of being stigmatized [5–7].

General practitioners (GPs) play a significant role in the detection, diagnosis, referral and treatment of depression, which is defined by a high point-prevalence of depressive episodes in primary care patients of 8–17% [8, 9]. This result was recently confirmed in an epidemiological study of primary care patients in Germany with a point-prevalence of 14.3%, according to self-reports [10]. Moreover, the majority of individuals with depression are being treated in primary care rather than in specialized care, while the treatment of depression according to guidelines depends heavily on a correct diagnosis (e.g. [4]). Studies in primary care settings have shown that only every second patient with depression is diagnosed correctly [10].

Several reasons for the false-negative detection of depression are discussed in the literature, such as heterogeneous depression symptoms, lacking objective laboratory markers, time restrictions and lacking reimbursements in primary care settings that impede the necessary exploration and evaluation for a differential diagnosis [10, 11]. Further, studies revealed that 44–69% of patients with depression report only their physical symptoms [12–14]. Other studies have shown that both a correct GP diagnosis and further adequate treatment rely heavily on the symptoms reported by the patient during consultation [10, 13, 15, 16]. The detection rate for individuals that report only physical symptoms is therefore much lower than for those that report physical and mental symptoms [15].

Different reasons for the association between depression and patients reporting mainly physical instead of mental complaints have been discussed, among them the stigma associated with the depression. Depression stigma might result in defense mechanisms such as masking certain symptoms and emphasizing somatic aspects so as to disguise mental health problems that may require treatment in anticipation of negative consequences [14, 16, 17].

Stigma is described as a discrediting attribute (e.g. physical attribute, religion, skin color, mental health disorder) by which the carrier deviates from the expected social norm [18]. Among the leading concepts of stigma is the one developed by Link and colleagues, describing stigma by use of the following five interrelated components: 1. labeling (creating labels for social salient attributes), 2. stereotyping (linking those labels to undesirable characteristics), 3. separating (the labeled, stigmatized group), 4. emotional reaction (of stigmatizers as well as stigmatized persons influencing their subsequent behavior), and 5. status loss and discrimination (of stigmatized persons) [19]. Another often cited concept of stigma was developed by Corrigan [6], who defined stigma with three core components of stereotypes, prejudices and discrimination, while distinguishing between two types of stigma: Public stigma, which refers to the (discriminating) reactions of the society towards a stigmatized person or group (e.g. withholding a job) based on stereotypes (e.g. “Individuals with depression are incompetent.”), and prejudices (believing in these stereotypes) [6, 20]. When public stigma is internalized by an individual belonging to the stigmatized group, it may result in self-stigma [6, 21, 22], thereby lowering the individual’s self-esteem [20, 23]. This process appears to be moderated by further factors, e.g. if an individual is conscious about the public stigma [24] and in agreement with public stigmatizing attitudes [20]. Two further related types of stigma have been found in the literature: Personal stigma, which describes a person’s attitudes toward a mental health disorder regardless of whether he/she belongs to the stigmatized group [7, 25, 26], and perceived stigma, which is related to public stigma and describes an individual’s belief about public attitudes towards a mental health disorder [6, 7, 26–28]. Some authors have used public and perceived stigma synonymously [7, 29]. Beside the different types and concepts of stigma, different measures exist to assess the various stigma types [19, 21].

Several studies and theories can be found in the literature describing the association between different stigma types and help-seeking for mental health disorders. Despite mixed results, there is evidence that different types of stigma can influence different stages of the help-seeking process (e.g. [7, 30, 31]). Self-stigma appears to be associated with less help-seeking intentions and behaviors at an early stage of the help-seeking process. Studies assessing self-stigma via the Self-Stigma of Seeking Help (SSOSH) scale [31, 32] or by the Internalized Stigma of Mental Illness (ISMI) scale [33] have also found that being labeled “mentally ill” may pose a potential threat to an individual’s self-esteem. Like self-stigma, there is some evidence that personal stigma may also impact help-seeking at an early stage, e.g. by not appraising symptoms as a mental health problem and a perceived need for professional help. Schomerus and colleagues [30] assessed personal stigmatizing attitudes by observing participants’ attitudes of blaming mentally ill people (according to the Self–Stigma of Mental Illness Scale (SSMIS) [34]), their discrimination of mentally ill people and the social distance they maintain towards them (Social Distance Scale, [35]). The authors concluded that participants who supported discrimination or blaming a person with a mental illness showed lower self-identification of having a mental health problem themselves, as well as a lower perceived need for help. Likewise, Griffith and colleagues reported that within a sample of 2,000 Australian adults, a positive association was found between personal depression stigma, assessed with the DSS [25, 27], and the belief to deal with depression alone [26].

While personal stigma has been shown to impair early stages of help-seeking, i.e. avoiding professional help, perceived and public stigma do not appear to have comparable effects on initial help-seeking [7, 17, 21, 22, 29, 30, 33]. Schomerus and colleagues [22] assessed a person´s beliefs about public attitudes towards seeking psychiatric help with use of the 17-item ADSP scale (anticipated discrimination when seeing a psychiatrist), revealing no association between beliefs and help-seeking intentions. Meanwhile, the authors also found that participant´s personal attitudes, assessed by their desire for social distance, decreased their help seeking intentions. A systematic review on active help-seeking and mental-health related stigma obtained similar results: public stigma, mainly assessed with the Perceived Devaluation Discrimination Scale [36] and its adaptations, was not linked to active help-seeking [7]. Similar results have been reported for perceived stigma, which was assessed with the Perceived Devaluation Discrimination Scale, and which was not found to be associated with barriers to depression care such as a low perceived need, negative treatment expectations or treatment seeking attitudes and behaviors [29, 33]. However, in these same studies, self-stigma, assessed by the 29-item Internalized Stigma of Mental Illness (ISMI) scale [37], showed a strong association with barriers to care and treatment seeking attitudes and behaviors respectively.

Some studies have indicated that perceived stigma may also be a relevant factor in later stages of the help-seeking process by impeding engagement in and maintenance of depression treatment as well as treatment outcomes [38–40]. However, the full scope of the influence of perceived stigma remains unknown.

The current study investigates depression stigma in a large sample of primary care patients in Germany with and without depression (based on self-reports) as well as their reported complaints to the GP (including type and number of complaints). To our knowledge, there are only two studies available that have investigated whether primary care patients with depression differed from primary care patients without depression in regard to the number of reported complaints to their GP [14, 41]. Both studies reported a higher number of physical complaints reported to the GP by patients with depression with a mean of 4.4 (*SD* = 4.2) and 4.5 (*SD* = 2.3) respectively, compared to patients without depression with a mean of 1.2 (*SD* = 1.9) and 1.8 (*SD* = 1.3) respectively.

Based on previous literature, we state the following hypotheses:

1. In line with previous findings regarding reported complaints to the GP, we assume that patients with depression will present more physical than mental complaints, as well as more symptoms in total, in comparison to patients without depression [14, 41].
2. Patients with depression will not differ in personal depression stigma from patients without depression, as personal depression stigma has been reported to deter help-seeking at an early stage (i.e. the wish to deal with the problem alone instead of seeking professional help, e.g. from a GP [26]).
3. With regard to perceived depression stigma, patients with depression will show higher stigma scores than patients without depression, as has been found in other studies [25, 41, 42].
4. Further, for the subgroup of patients with depression, we will explore to which degree perceived depression stigma is relevant at this stage of the help seeking process (consulting a GP). This will be determined through associations between the number and types of reported complaints for consultation (mental vs. physical), as well as the factors related to the treatment of depression (type of treatment, help-seeking behaviors and/or referral to specialized care).

## Materials and methods

### Sample and procedures

**Fig 1:**
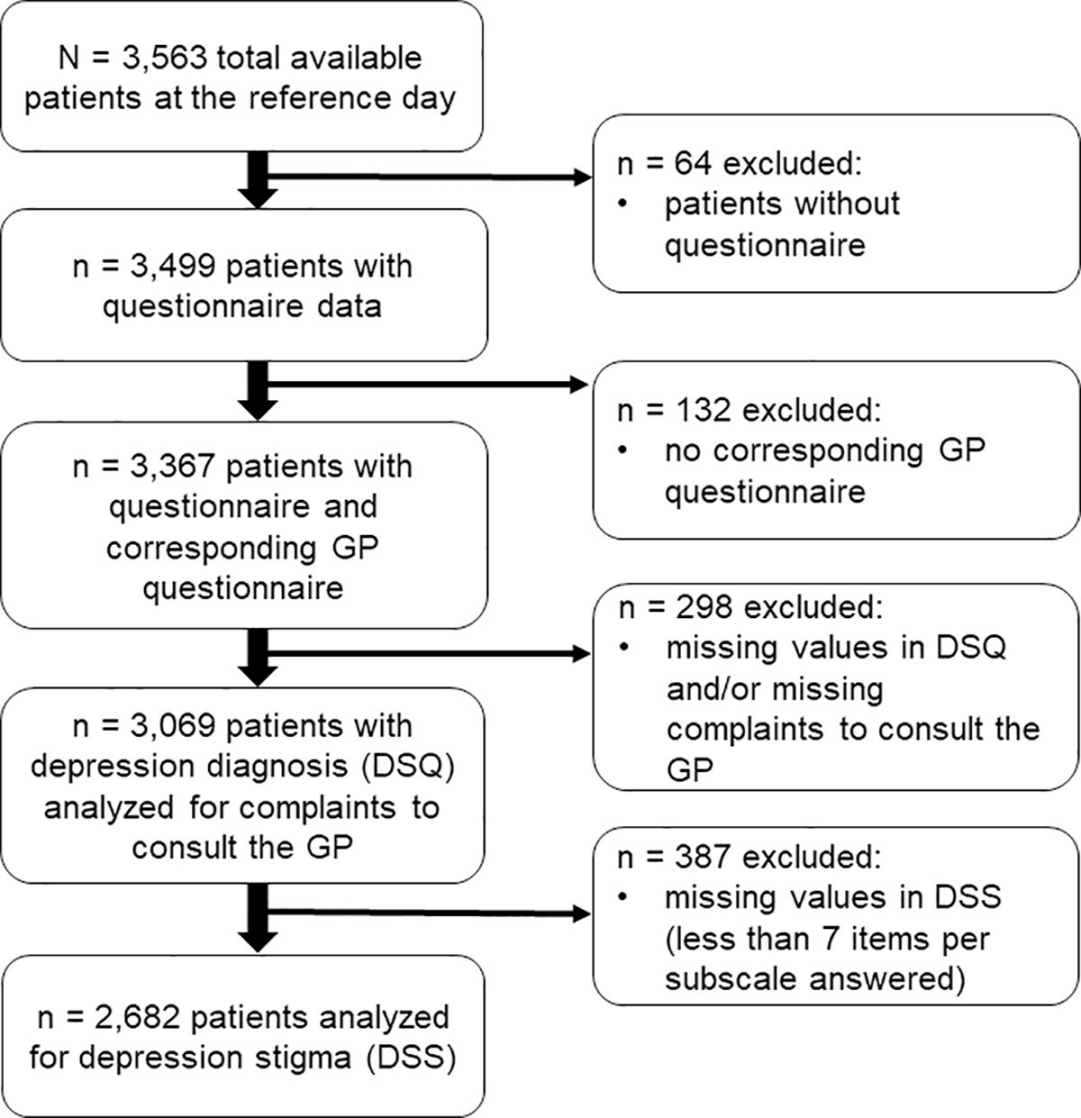
Flow chart of sampling process.

As part of a cross-sectional epidemiological study investigating the diagnosis and treatment of depression in the primary care setting in Germany (VERA study), 269 randomly selected GPs of six regions in Germany (Dresden, Leipzig, Frankfurt/ Kassel/ Fulda, München, Berlin, Hamburg) stratified by location (city, town, rural) were recruited to participate in a survey at the end of 2013 and the beginning of 2014 (response rate 5.8%). These regions were selected based on their representativeness for the heterogeneous geographical situation within the federal territory of Germany. In total, 253 GPs and 3,563 unselected patients (response rate 55.9% of suitable patients) took part in the survey by completing a GP and a patient questionnaire, respectively. The patient questionnaire consisted of a compulsory Part A and a voluntary Part B. Part A contained the Depression-Screening-Questionnaire (DSQ; [43]) and asked patients to state the complaints for which they consulted their GP. Meanwhile, Part B contained the Depression Stigma Scale (DSS; [27, 28]). As illustrated in Fig 1, 64 of the 3,563 unselected patients were excluded (no patient questionnaire available), resulting in 3,499 patients with questionnaire data suitable for analysis. Another 132 cases were excluded because there was no corresponding GP questionnaire. Of the remaining 3,367 patients, 298 cases were excluded due to missing data in the DSQ items and/or items to assess complaints to consult the GP for this analysis. Therefore, the final sample in which we analyzed complaints reported when consulting a GP with regard to depression diagnosis consisted of 3,069 patients. Of these patients, 387 cases were excluded due to missing data in the DSS. Depression stigma analysis were therefore performed for a sub sample of 2,682 patients (see results section). A detailed description of the design of the VERA study can be found elsewhere [4, 10].

Written informed consent was obtained prior to study participation from all participating GPs and patients. The study was approved by the ethics committee of the Technische Universität Dresden on 2013.10.07 under the reference number EK 392102013 and according to the Declaration of Helsinki.

### Instruments

#### Sociodemographic data

Sociodemographic information including age (in years), gender, marital and occupational status was collected. Marital and occupational status was dichotomized into “single” vs. “not single” and “occupied” vs. “not occupied”, respectively.

#### Depression diagnosis

Depressive symptoms were rated by patients according to ICD-10 criteria using the Depression-Screening-Questionnaire (DSQ; [43]). In total, 12 items regarding depressive symptoms during the preceding 2 weeks were rated on a three-point Likert-scale (0 = “not at all”, 1 = “sometimes”, 2 = “most of the days”). If at least 3 items were endorsed with “most of the days” and the sum score was higher than 7 (i.e. at least 4 symptoms were present), the study diagnosis “depression” was coded (according to [44]). Depression severity was rated as follows: “mild depression” if at least 3 items were indicated for “most of the days” and the sum score exceeded 7, “moderate depression” if at least 5 symptoms were indicated for “most of the days” and the score exceeded 11, and “severe depression” if at least 7 symptoms were indicated for “most of the days” and the score exceeded 16 (according to [4]). Depression severity was dichotomized into “mild” vs. “moderate or severe”. The DSQ demonstrated good internal consistency (Cronbach’s alpha of 0.83) as well as high inter-rater reliability (kappa = 0.84–0.89) in a German study with primary care patients [45].

#### Complaints reported to the GP

Patients provided information regarding the complaints they reported during their consultation with a GP through use of a multiple-choice questionnaire containing seven response categories (physical complaints or illnesses; sleeping problems; pain; depression or depressiveness or desperation; anxiety problems; other mental health problems; another reason). Multiple answers were possible. For the category “another reason”, follow up appointment and referral due to any emergency were given without asking for the type of complaint. “Physical complaints” were counted if a patient indicated a physical complaint or illness, sleeping problems or pain as the reason they consulted the GP. “Mental complaints” were counted if a patient indicated they sought help from a GP for either depression, depressiveness or desperation, anxiety problems or other mental health problems. “Physical and mental complaints” were counted if a patient indicated at least one reason from each of the aforementioned categories. The items used to assess complaints reported to the GP were developed by the researchers (psychologists, senior psychiatrists and GPs), as there was no established instrument available.

#### Depression treatment and treatment-related factors

Patients who endorsed at least 2 DSQ items with either “sometimes” or “most of the days” were asked to document their current and planned future treatment of depression. In addition, GPs provided information for each patient regarding the type of treatment he/she had received prior to the reference date (i.e. the day when the questionnaires were completed), as well as any other treatment of depression. In order to maximize sensitivity in identification, treatments were assumed to be present if they were mentioned by the patient and/or the GP. Treatment was categorized according to Trautmann [4] into (1) psychotherapy, (2) antidepressants, (3) other treatment, and (4) no treatment. According to the National Disease Management Guidelines, Unipolar Depression, the evidence-based recommendations for diagnosis and treatment of unipolar depression in Germany [46] with either psychotherapy or antidepressants are indicated to treat mild to moderate depression, whereas a combination of both is indicated in the case of severe depression. For a more detailed description of the categorization of treatments, please refer to Trautmann [4] (supplementary material, eTable 1).

To assess referral to and/or help-seeking from specialized care (i.e. psychiatrist, psychotherapist, inpatient treatment), patients were asked if their GP had referred them to specialized care or if they had sought specialist help by themselves due to their depressive symptoms.

#### Depression stigma

Attitudes towards depression were assessed by the standardized Depression Stigma Scale (DSS), a commonly used instrument to assess depression stigma in the general public as well as depressed individuals [27, 28]. The DSS measures perceived and personal stigma with 18 items which are scored on a five-point Likert Scale ranging from 1 = “strongly disagree” to 5 = “strongly agree” [28]. Items cover common prejudices including depression as weakness of character or personal fault, unpredictability and dangerousness, shame, avoidance, and discrimination. For nine items the participant indicates to what extent he/she agrees with a statement reflecting personal depression stigma (e.g. “Depression is a sign of personal weakness.”). In the remaining nine items, the participant rates what he/she thinks the broad public believes about these same statements (e.g. “Most people believe that depression is a sign of personal weakness”), thereby reflecting perceived depression stigma. Higher sum scores on each subscale (range 9–45) and as a total score (range 18–90) indicate more stigmatizing attitudes. The DSS has shown high test-retest reliability as well as moderate to high internal consistency across various countries and in different populations (Cronbach´s alpha ranging from .70 - .82 for subscales and total scale) [25, 28, 47–49]. We used the German version of the DSS [49], which has been translated both forward and back from the original English DSS in accordance with the guidelines of the World Health Organization [50] by a native German speaker and a German mental health professional. The DSS factor-structure depends on language, sample and cultural context and was subject to previous studies [48, 51–54]. To date, the factor-structure of the German version of the DSS has not been investigated.

### Statistical analysis

Statistical analyses were performed using IBM SPSS Statistics version 25.0. A two-tailed α = 0.05 was applied to statistical testing. First, to examine group differences between patients with and without a depression diagnosis, χ^2^ tests were used for categorical variables (gender, marital status, occupational status and reported complaints). Bonferroni correction was applied for post-hoc analysis in case of multiple tests. Group differences for continuous variables were analyzed using Mann-Whitney *U* tests, as all continuous outcome variables (age, number of reported complaints per patient, DSS sum score and subscale scores) were non-normally distributed, as indicated by the Shapiro-Wilks test (all *p* < .05). Additional exploratory analyses of covariance (ANCOVAs) examined group differences in DSS sum scores and subscale scores, respectively, when adjusting for age, gender and marital status.

Second, in order to examine whether sociodemographic and treatment-related factors predicted DSS perceived stigma scores (dependent variable) in the subgroup of patients with depression, a multiple linear regression analysis was applied with the following predictor variables: age, gender, severity of depression (according to self-report DSQ), number and type of complaints reported to the GP, help-seeking from specialized care and depression treatment. Categorical variables with more than two categories (depression severity, complaints and depression treatment) were recoded into binary dummy variables. The variable “treatment according to guidelines”, which combines the type of depression treatment and depression severity, was initially included in the regression analysis but did not become significant. Therefore, depression severity and treatment were included as separate predictors since their potential association with perceived stigma is of great practical interest. All predictor variables were entered simultaneously. The dummy variables for “no treatment” and “reporting only physical complaints to the GP” had to be excluded from the regression analysis due to multicollinearity. All effect sizes were interpreted as suggested by Cohen [55], i.e. 0.2 was considered a small effect, 0.5 a medium, and 0.8 a large effect.

## Results

### Sample

At the reference date, 430 out of 3,069 patients (14.0%) reported a current depressive episode according to the ICD-10 criteria, i.e. they received a depression diagnosis based on the DSQ. Of these patients, 261 (60.7%) reported a mild depressive episode, 114 (26.5%) reported a moderate depressive episode and 55 (12.8%) reported a severe depressive episode. Differences between patients with (*n* = 430) and without (*n* = 2,639) depression with regards to sociodemographic information, depression stigma and complaints reported to the GP are displayed in Table 1. All analyses of depression stigma were conducted for a reduced sample due to missing data: 2,357 (76.8%) participants of the total sample (3,069) answered all DSS items without missing values. For 712 patients with missing values in the subscales, data was computed by the patient´s subscale mean score for non-missing items if the patient answered at least 7 out of 9 (78%) subscale items according to Dardas [56]. Thus, 325 patients with missing data met these criteria for data imputation, resulting in a total sample of 2,682 patients with DSS scores.

**Table 1:** Sociodemographic characteristics, depression stigma and complaints reported to the GP in patients with and without depression.

	Patients with depression^a^ (*n* = 430)	Patients without depression (*n* = 2,639)	Test	Effect size
	*N* (%) or *M* (*SD*)	*N* (%) or *M* (*SD*)		
Age (years)	50.22 (15.81)	53.9 (17.12)	*U* = 489,994.50	*r* = .08
			*p* < 0.01	
Gender^b^				
Male	137 (31.9%)	1,061 (40.2%)	χ^2^ (1) = 10.89	φ = .06
Female	293 (68.1%)	1,576 (59.8%)	*p* = .001	
Marital status^b^				
Not single	210 (49.8%)	1,561 (60.0%)	χ^2^ (1) = 15.73	φ = .07
Single	212 (50.2%)	1,040 (40.0%)	*p* < .001	
Occupational status^b^				
Occupied	254 (60.2%)	1,572 (60.0%)	χ^2^ (1) = .03	φ = .00
Not occupied	168 (39.8%)	1,020 (39.3%)	*p* = .858	
Number of reported complaints per patient	2.02 (1.33)	1.2 (0.69)	*U* = 362,665.50	*r* = .25
			*p* < .001	
Type of complaints reported to the GP	430 (14%)	2,639 (86%)	χ^2^ (4) = 449.81	φ = .38
			*p* < .001	
Number of patients only stating physical complaints	190 (42.2%)	1560 (59.1%)	χ^2^ (1) = 33.62	φ = .11
			*p* < .001	
Number of patients only stating mental complaints	42 (9.8%)	59 (2.2%)	χ^2^ (1) = 65.90	φ = .15
			*p* < .001	
Number of patients stating physical and mental complaints	124 (28.8%)	93 (3.5%)	χ^2^ (1) = 360.57	φ = .34
			*p* < .001	
Number of patients stating no complaints	19 (4.4%)	213 (8.1%)	χ^2^ (1) = 7.06	φ = .05
			*p* = .008	
Number of patients with other reasons^c^	55 (28.8%)	714 (27.1%)	χ^2^ (1) = 40.07	φ = .11
			*p* < .001	
Depression stigma^b^	*Median* (*IQR*)	*Median* (*IQR*)		
DSS sum score	48 (40–54)	46.3 (29–53)	*U* = 420,759.50	*r* = .00
			*p* = .047	
DSS personal stigma	20 (16–24)	20.25 (16.9–25)	*U* = 441,133.00	*r* = .03
			*p* = .118	
DSS perceived stigma	27 (21–32)	25.9(20–29)	*U* = 399,821.50	*r* = .08
			*p* < .001	

*N* = Number of patients; % = percent calculated for valid cases; *M =* mean; *SD =* standard deviation; DSS = Depression Stigma Scale.

^a^ according to DSQ self-report.

^b^ reduced sample size due to missing data, valid percentage are reported.

^c^ follow up appointments and emergency cases

Patients with depression were significantly younger, more likely to be female, more frequently single (small effects, all *p* < .01) and reported an average of 2 (vs. 1) complaints to their GP (small effect, *p* < .001), when compared to patients without depression. Regarding the complaints reported to the GP, 190 (42.2%) patients with depression reported physical complaints only, 42 (9.8%) reported mental complaints only and 124 (28.8%) consulted the GP due to mental and physical complaints. Patients with depression significantly differed in all 3 categories from patients without depression (small to medium effects, all *p* < .001, see Table 1).

Further, both subsamples differed in perceived depression stigma scores and depression stigma sum scores, with higher scores in patients with depression (small effect, *p* < .001 and *p* = .047). No differences were found for occupational status and personal depression stigma scores (*p* > .05). Three additional exploratory ANCOVAs with DSS sum scores and subscale scores as outcome variables did not result in changes in the significance of results when including age, gender and marital status as covariates.

Results of an exploratory linear regression analysis for the subgroup of patients with depression are presented in Table 2.

**Table 2:** Linear regression for predictors of perceived depression stigma.

	DSS perceived stigma score (*n* = 391^a^)					
Variable	Unstan-dardized β	SE	Standar-dized β	95% Confidence Interval (CI)	*t*	*p*
Age	-.07	.04	-.10	-.141, .010	-1.70	.091
Gender	-3.53	1.24	-.14	-5.993, -1.062	-2.81	**.005**
Depression severity ^b^	-.13	.87	-.01	-1.844, 1.582	-.15	.880
Number of reported complaints to consult the GP	1.27	.71	.14	-.127, 2.667	1.79	.075
Number of patients only reporting mental complaints	1.97	2.09	.05	-2.130, 6.072	.95	.345
Number of patients reporting physical and mental complaints	.62	1.92	.02	-3.148, 4.386	.32	.75
Number of patients with different reasons^c^	2.24	1.95	.06	-1.585, 6.073	1.15	.25
Number of patients reporting no complaints	3.23	3.14	.06	-2.936, 9.393	1.03	.304
Only pharmacological treatment	-4.97	2.15	-.14	-9.189, -.747	-2.31	**.021**
Only psychotherapeutic treatment	-3.65	2.02	-.11	-7.617, .314	-1.81	.071
Combination treatment	-3.41	1.86	-.12	-7.086, .247	-1.83	.068
Any treatment other than pharmacological or psychotherapeutic	-1.64	1.66	-.06	-4.903, 1.625	-.99	.324
Referral to and/or help-seeking from specialised care	-.06	1.30	-.01	-2.613, -2.503	-.04	.966
*R*^2^ (*R*^2^ adjusted)	.06 (.03)					
*F*	1.94					
*p*	= .025					

^a^ reduced sample size due to missing data.

^b^ depression severity dichotomized into “mild” vs. “moderate or severe”.

^c^ patients in emergency cases, coming for referral, prescription, or follow-up appointment only.

Male gender (*p* = .005) and less frequent pharmacological treatment (*p* = .021) predicted higher scores of perceived depression stigma in patients with depression. The other predictors were unrelated to perceived depression stigma (all *p* > .05). The overall model fit was *R*^2^ = 0.06 (adjusted *R*^2^ = 0.03).

## Discussion

Almost half of the primary care patients with depression reported mostly physical complaints to their GP. Compared to patients without depression, patients with depression reported significantly more complaints, as well as a more frequent combination of physical and mental complaints. Patients with depression did not differ from patients without depression regarding personal depression stigma but showed higher perceived depression stigma and therefore higher stigma scores in total.

Patients with depression presented significantly less exclusively physical complaints to their GP than patients without depression. Nevertheless, 40% of patients with depression did not report any mental health symptoms to their GP. Only 1 out of 10 patients with depression indicated that they consulted their GP due to (only) mental complaints (according to hypotheses 1). In comparison, one third of patients with depression reported a combination of mental and physical complaints. The large proportion of patients with depression who reported only physical complaints is aligned with findings of previous studies, including a study of German primary care patients with depression, wherein 57% of patients reported only somatic symptoms [13], as well as an international study in which a range of 45–95% of primary care patients with depression exclusively reported somatic symptoms to their GP [14]. Further, the combination of different complaints, i.e. mental and physical symptoms, appears to be an important indicator for depression, as patients without depression reported this combination of symptoms significantly less frequently than patients with depression. In addition, depressive patients reported twice as many complaints in total during their consultation with the GP, in comparison to patients without depression. Our results thereby provide further evidence for this phenomenon which has previously been reported by only a handful of studies with comparable ratios [14, 41].

The relevance of patients with depression reporting mainly physical symptoms, as well as their nondisclosure of mental symptoms has been widely discussed in the literature. Contributing factors to this phenomenon may include: attributing depressive symptoms to somatic causes [15, 57], believing somatic symptoms to be a core component of depression, a lack of trust in primary care providers regarding the care of depression, fear of being placed on antidepressants, as well as stigma surrounding depression [14, 16, 58, 59].

In our sample, personal depression stigma was comparable between patients with and without depression (according to hypothesis 2). Prior studies investigating path models of help-seeking concluded that personal stigma may affect help seeking behavior at an early stage, e.g. recognizing and appraising symptoms, a perceived need for help [7, 30, 60], and the desire to deal with the problem alone [7, 26, 30]. This implies that the current study may have predominantly investigated patients with comparably low personal depression stigma, since these patients may have identified or recognized their symptoms as being related to a mental illness or else perceived a need for treatment which was followed by the intention and respective action to seek help, which in our study was the consultation with a GP.

Perceived depression stigma was significantly higher in patients with depression as compared to patients without depression (according to hypothesis 3), which is similar to the results of previous studies measuring perceived stigma with the corresponding DSS subscale [25, 41, 42] and the Stigma Scale for Receiving Psychological Help (SSRPH) [41]. This suggests that individuals with depression may experience stigmatization from their community due to their diagnosis or, in case of first-time help seeking, are more sensitive to such events [22, 25].

It is also conceivable that the choice of primary care setting could have been influenced by patients´ perceived stigma, as consulting a GP does not provide the same level of branding someone as mentally ill as is associated with the consultation of other mental health specialists [6, 17, 21, 61]. On the other hand, accessing a GP in Germany is much easier compared to specialized care, especially in rural areas where the density of mental health professionals is comparably low. Thus, an appointment in primary care can usually be arranged at short notice and without waiting time. As we did not assess patients´ reasons to choose primary care providers, no conclusions on perceived stigma as a potentially influencing factor on the choice of care provider can be drawn from this study.

Higher scores of perceived stigma were unrelated to the type of complaints reported to the GP in our study, i.e. fearing negative consequences of a depression diagnosis by others did not correlate with the reporting of predominantly physical symptoms or the non-disclosure of mental symptoms in primary care settings. Simon [14] reported that in a sample of primary care patients with depression, the majority of patients reporting only somatic complaints (60%) did not deny depressive symptoms when asked. The authors argued that reporting physical symptoms to a GP might function as an “admission ticket” to primary care, as GPs are not commonly seen as the appropriate person to talk to about depression symptoms [16, 58, 62, 63]. In this case, perceived stigma plays a minor role with regards to complaints presented to the GP.

Examining the treatment for depression within our sample, patients with depression and higher scores of perceived stigma were less frequently treated with psychopharmacological medication. Our findings match those of previous studies, which reported lower initiation of pharmacological treatment and lower antidepressant medication adherence for individuals with depression who reported higher perceived stigma [21, 38, 40], as assessed with the Perceived Devaluation Discrimination Scale [36]. Since the assessment of perceived stigma was different and given the low percentage of variance explained by the regression model in our study, conclusions should be drawn with caution if and to what extent the fear of belonging to a stigmatized group when receiving pharmacological treatment for depression conflicted with the need for treatment [38]. Nevertheless, this finding is particularly important for patients with severe depression, as antidepressant medication is typically recommended for this group, either independently or in combination with psychotherapy.

Findings in the literature have indicated either no consistent gender differences in perceived stigma (e.g. [25, 39], higher perceived stigma in females [25, 42, 47, 56], or higher perceived stigma in males [64]. However, former studies on gender differences have been heterogenic regarding samples, cultural context and measures used to investigate stigma [64]. As a result, the conclusion of our study that male primary care patients with depression are more likely to be influenced by public attitudes than female patients requires further research. Nevertheless, perceived depression stigma may have an effect on medication adherence [40]. Therefore, GPs should pay special attention to male patients so as to address and manage the anticipated negative effects of treatment.

Perceived stigma is only one type of mental health stigma and help-seeking is a complex process that is influenced by a variety of different factors, including patient and illness characteristics [25]. Perceived stigma does not appear to prevent primary care patients from consulting a GP, even if we cannot yet draw conclusions on whether a patient associates his/her complaints with a mental health disorder or with depression. The question of whether perceived stigma influences the types of complaints reported, as well as how many complaints are reported by patients in primary care settings requires further research. In later steps of the help-seeking process, perceived stigma may hamper the treatment of depression, in particular pharmacological treatment and treatment for male patients. This should therefore be addressed in future studies.

## Conclusion

This study provides evidence for the importance of physical complaints reported by patients with depression in primary care settings, in light of the small number of patients disclosing mental symptoms. The study further emphasizes the importance of the number of complaints reported by patients as a potential marker for a depression. GPs should take note of patients reporting multiple complaints, particularly if this includes a combination of physical and mental complaints, and consider screening these patients for an underlying depression. Further to this, we suggest GPs should also address patients´ concerns about public attitudes towards depression and provide support to overcome them [11], particularly as pharmacological treatment may be influenced by the anticipation of negative consequences [38]. Broad awareness campaigns should include a focus on primary care settings as appropriate sources for depression care and individuals should be encouraged to disclose mental symptoms within this setting [16].

Future stigma research should focus on the different types of stigma, as well as the various stages of the help-seeking process. These studies should also take covariates into account (e.g. awareness of services, believing in treatment efficacy [31], and believing in a continuum of symptoms from health to illness [65]), so as to tailor individual interventions according to the respective stage of an individual’s help-seeking process.

### Strengths and limitations

Our study provides further evidence for the importance of the number of complaints primary care patients with depression report to their GPs. These findings are of great practical relevance, as patients with and without depression differ significantly in how many complaints they report. To date, there have been only two studies making mention of this phenomenon. Further, to our knowledge there has been no prior research regarding the association between the number and the type of complaints reported (physical vs. mental) and their association with perceived depression stigma. However, this study may also have had a number of limitations. Due to the cross-sectional design of the study, no causal inferences can be drawn. Further, as participation in the study was voluntary, a selection bias may have occurred and as a result, patients with lower stigma may have been overrepresented in our sample. Social desirability bias when answering the DSS might also have been an issue, resulting in an underestimation of depression stigma within our sample. Moreover, the factor structure of the German version of the DSS has not been replicated and its psychometric properties require further research. Depression diagnosis was based on self-report assessments with the DSQ, and a potential bias may have occurred. We did not include GP diagnoses in our analyses, as this could have significantly reduced our sample size. Further, from a clinical point of view, the patients’ subjective condition was our main area of interest. Since we did not assess the specific type of complaints for patients who self-referred to the GP for immediate care (i.e. emergency case) or for follow-up appointment, a potential bias cannot be ruled out. Finally, when predicting perceived stigma we were not able to control for all potential influencing factors (e.g. mental health literacy) in a systematic way.

## Supporting information

S1 Dataset Study data to reproduce the results. (XLSX)
